# Regional Profile of Food Allergen Sensitization Among Children in Southwest China: A Cross-Sectional Study

**DOI:** 10.3390/jcm15052032

**Published:** 2026-03-06

**Authors:** Lingyi Yan, Menglan Zhang, Chenxi Liu, Yifei Duan, Yu Wu, Qinni Yang, Zhengxiang Gao

**Affiliations:** 1 Department of Laboratory Medicine, West China Second University Hospital, Sichuan University, Chengdu 610041, China; 2 Key Laboratory of Birth Defects and Related Diseases of Women and Children, Sichuan University, Ministry of Education, Chengdu 610041, China

**Keywords:** allergen, food allergy, epidemiology, public health, children, China

## Abstract

**Background/Objectives**: As an escalating global health challenge, food allergies impose substantial burdens on the physical and psychological well-being of pediatric populations, profoundly compromising their quality of life. Given the marked geographical heterogeneity in allergen distribution patterns, this epidemiological investigation systematically characterizes prevalent pediatric food allergens sensitization patterns in Southwest China, yielding critical region-specific data to inform targeted prevention strategies and clinical management protocols. **Methods**: A cohort of 36,399 pediatric participants (age <18 years) underwent hospital-based testing for allergen-specific immunoglobulin E (sIgE) reactivity against 10 regionally prevalent food allergens, utilizing a semi-quantitative, immunocapture-based, and enzyme-linked immunosorbent assay. **Results**: Of the 36,399 children whose food allergen sensitization profiles were analyzed, 48.12% (*n* = 17,514) demonstrated elevated sIgE reactivity in response to at least one tested allergen, with milk and eggs emerging as the predominant allergenic triggers. Age-stratified analyses identified preschool age (≤6 years) as the critical window for food sensitization, demonstrating peak IgE reactivity to major allergens, including milk, peanuts, soybeans, shrimp, eggs, wheat, and beef. Male subjects exhibited significantly higher sensitization rates to peanuts, soybeans, crustaceans and wheat compared to females (*p* < 0.05), underscoring the importance of sex-based considerations in allergy prevention strategies. **Conclusions**: Milk and eggs emerge as the dominant food allergens that cause sensitization in Chengdu’s pediatric population. Age- and sex-dependent vulnerabilities were identified, with younger children and male participants demonstrating higher sensitization rates than their counterparts. These age-stratified and male-predominant sensitization patterns provide a scientific foundation for public health initiatives.

## 1. Introduction

Food allergies (FAs) now affect approximately 10% of the population and have demonstrated a concerning upward trajectory over the past two to three decades, emerging as a pressing public health challenge worldwide [1,2,3]. IgE-mediated food allergies typically emerge during infancy and frequently co-occur with eczema as one of the earliest clinical manifestations of the atopic march. This early allergic predisposition substantially increases the risk of subsequent asthma and other IgE-related disorders in later developmental stages [4]. Beyond its physiological consequences, pediatric food allergies impose significant psychological distress on affected children while adversely impacting their overall health-related quality of life. Moreover, this condition places a considerable psychosocial and economic burden on their families [5,6].

Epidemiological evidence demonstrates that the most common food allergens include peanuts, tree nuts, milk, eggs, fish, shellfish, wheat, soy, and seeds [7]. A comprehensive global survey conducted by the World Allergy Organization (WAO) encompassing 89 member states revealed striking epidemiological heterogeneity in childhood food allergy prevalence rates. In infants and preschool children (<5 years), the prevalence of food allergies ranges from 1% in Thailand to 10% in Australia [8]. Furthermore, peanut allergy rates are significantly higher in Northern Europe, North America (the United States and Canada), and Australia compared to Southern/Eastern Europe and Asia [9,10]. In China, the primary allergenic foods are eggs, milk, wheat, crustaceans (particularly shrimp), shellfish, and fish, with mango representing the predominant fruit allergen, while peanut allergies exhibit notably low prevalence rates (<1% incidence) compared to Western populations [11,12]. In addition, sesame and camel milk allergies are more common in the Middle East than in other areas [13,14]. Given the considerable geographic heterogeneity in allergen profiles, targeted screening for regionally prevalent allergenic triggers serves dual purposes: (1) generating population-specific epidemiological data to inform public health interventions and (2) enabling clinicians to adopt a stratified diagnostic approach that prioritizes locally dominant allergens. This synergistic strategy optimally bridges evidence-based clinical guidelines with regional immunological realities. Research on allergen sensitization patterns among children in Southwest China remains insufficient, particularly for studies employing large sample sizes.

The aim of this population-based cross-sectional investigation was to systematically characterize the regional sensitization profile of predominant food allergens while evaluating age-stratified and sex-specific variations in reactivity patterns.

## 2. Materials and Methods

### 2.1. Study Design

This was a hospital-based, retrospective cross-sectional study based on anonymized data from 36,399 pediatric patients (<18 years old) who underwent specific IgE (sIgE) screening for common food allergens at the West China Second Hospital of Sichuan University. This project took place over a two-year period, spanning 1 January 2023 to 31 December 2024, and was meticulously documented.

### 2.2. Participants and Data Collection

The study cohort included three distinct subgroups: (1) asymptomatic individuals undergoing preventive health screenings, (2) patients with self-reported allergic manifestations, and (3) clinically evaluated cases referred for specific IgE testing based on physician-assessed symptom profiles.

The following exclusion criteria were applied to ensure data quality and homogeneity: (1) age ineligibility—individuals aged ≥18 years at recruitment; (2) incomplete data—records with missing or severely incomplete key variables, including a lack of basic demographic information (e.g., age, sex) or missing or uninterpretable sIgE test results; and (3) duplicate testing—for subjects with who underwent multiple tests during the study period, only the first test record was included.

Basic demographic information (age, sex, and visit dates) was systematically recorded. It should be noted, however, that formal clinical diagnoses were not recorded. Moreover, no confirmatory clinical challenges or additional diagnostic validation procedures were conducted during the study period.

This investigation was conducted in strict compliance with the ethical principles outlined in the Declaration of Helsinki, with prior approval obtained from the Institutional Review Board of the West China Second University Hospital.

### 2.3. Serum sIgE Test

Serum allergen-specific immunoglobulin E (sIgE) concentrations were quantified using the Fooke^TM^ automated immunoassay platform (HOB Biotech Group Corp., Ltd., Suzhou, China), employing a proprietary semi-quantitative, immunocapture-based, and enzyme-linked immunosorbent assay (ELISA) in accordance with standardized protocols. All tests employed in this study were conventional screening tests based on allergen extracts. Our comprehensive allergen panel focused on predominant food allergens endemic to the region, including bovine milk, chicken eggs, peanuts, soybean, crabs, shrimp, wheat, codfish, beef, and mutton. The system classified sensitization levels semi-quantitatively according to established clinical thresholds: level 0 (<0.35 IU/mL), level 1 (possible or mild sensitization, ≥0.35 and <0.70 IU/mL), level 2 (mild sensitization, ≥0.70 and <3.50 IU/mL), level 3 (moderate sensitization, ≥3.50 and <17.50 IU/mL), level 4 (moderate to severe sensitization, ≥17.50 and <50.00 IU/mL), level 5 (severe sensitization, ≥50.00 and <100.00 IU/mL), and level 6 (extremely severe sensitization, ≥100.00 IU/mL). Although the standard diagnostic threshold for allergen sensitization is typically set at 0.35 IU/mL (level 1), this cutoff value exhibits high sensitivity but a relatively poor specificity in predicting clinically relevant allergies. Prior to conducting the test, we verified the performance of both the kit and the instrument. This verification assessed parameters including the detection limit, precision, carryover contamination rate and clinical concordance, following the quality specifications of the International Organization for Standardization (ISO) 15189 [15] for medical laboratories. The laboratory also performs daily internal quality control with manufacturer-supplied materials. Furthermore, it participates successfully in the external quality assessment scheme administered by the National Clinical Research Center for Dermatologic and Immunologic Diseases (NCRC-DID) in China. These measures ensure the ongoing accuracy of results and their comparability across laboratories.

### 2.4. Statistical Analysis

All statistical analyses were performed using the Statistical Package for Social Sciences version 23.0 (IBM SPSS Statistics for Windows; IBM Corp., Armonk, NY, USA) following standard biostatistical protocols. Categorical variables were presented as frequency counts (percentages). Between-group comparisons employed chi-square tests (for categorical variables with expected frequencies >5) or Fisher’s exact tests (for categorical variables with expected frequencies ≤5), with statistical significance defined a priori as *p* < 0.05 (two-tailed). Given the exploratory and descriptive nature of this study, we did not apply a formal correction for multiple comparisons, such as the Bonferroni method. This approach was taken to minimize the risk of Type II errors. It also helps prevent the potential masking of meaningful patterns in the data.

## 3. Results

### 3.1. Characteristics of the Study Population

This retrospective analysis evaluated the allergen sensitization profiles of 36,399 pediatric patients (56.23% male, 43.77% female; male/female ratio 1.24:1) that presented to our clinic. The subjects were stratified according to key developmental stages: infants (>0 and ≤1 year; *n* = 7538), toddlers (>1 and ≤3 years; *n* = 14,238), preschoolers (>3 and ≤6 years; *n* = 8605), school-aged children (>6 and ≤14 years; *n* = 5047), and adolescents (>14 and ≤18 years; *n* = 971). Notably, toddlers represented the largest patient subset (39.12% of total cohort), likely reflecting both increased allergy symptom emergence and routine screening practices during this developmental period. Our epidemiological assessment revealed distinct seasonal variations in the testing frequency, with peak diagnostic evaluations occurring during spring and summer months (March–August) (Table 1).

### 3.2. Allergen Sensitization Prevalence Patterns

**Figure 1:**
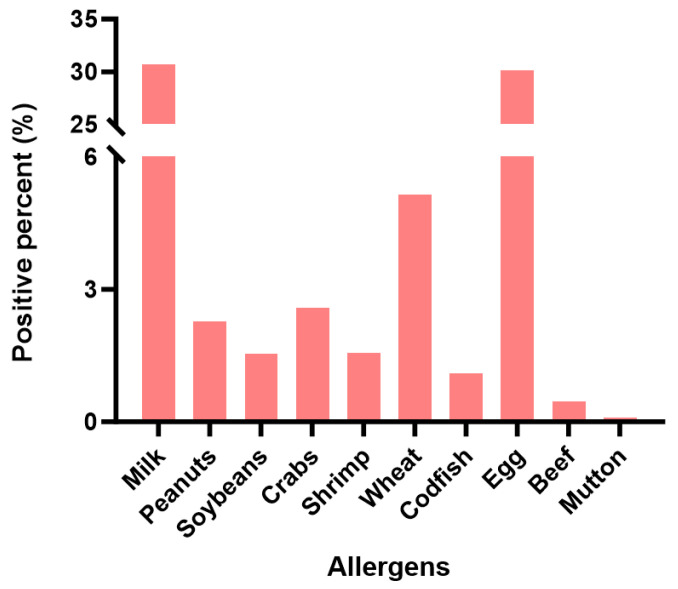
The detail allergen sensitization frequencies.

Our analysis revealed distinct allergen sensitization patterns across the study population. In total, 48.12% (17,514/36,399) of patients were reactive to at least one food allergen. Among these, milk emerged as the predominant allergen, exhibiting the highest sensitization frequency (31.18%), followed by chicken eggs (30.57%) as the second most prevalent allergen. In contrast, mutton (0.20%) demonstrated the lowest sensitization rates among the food allergens tested, marginally surpassed by beef (0.58%). Detailed allergen sensitization frequencies across the study population are illustrated in Figure 1.

**Figure 2:**
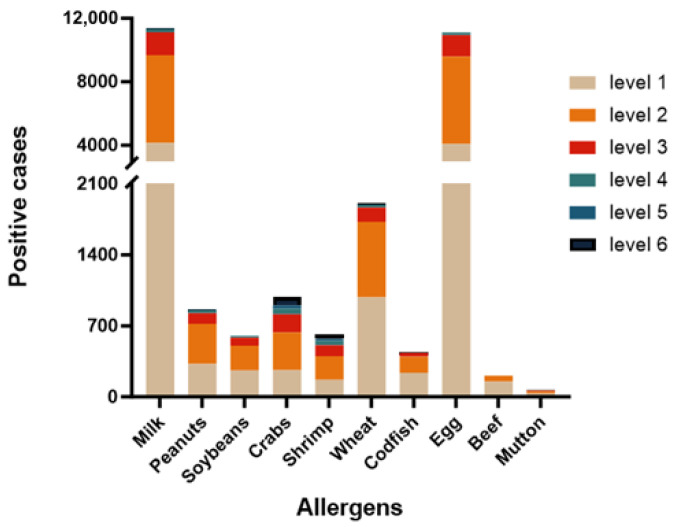
The positive cases to different allergens.

Our quantitative analysis revealed that sIgE reactivity was predominantly concentrated within the mild to moderate sensitization grades (level 1–3). Severe sensitization (level 5–6) accounted for a comparatively small proportion of cases (Figure 2).

### 3.3. Age Differences in Allergen Sensitization Profiles

Our descriptive analysis identified distinct age-dependent sensitization profiles, with the highest prevalence of milk (44.34%), peanut (2.93%), soybean (2.54%), shrimp (2.16%), and codfish (1.78%) specific IgE positivity occurring in children aged 1–3 years. Among preschool children (3–6 years), eggs (43.31%), wheat (7.89%), and beef (0.78%) demonstrated peak sensitization rates, while crab and mutton exhibited maximal reactivity in children older than 6 years of age (Table 2).

The China Consensus Document on Allergy Diagnostics provides a detailed delineation of the sIgE concentration, grade, level and clinical significance [16]. Within this standardized framework, grades 4–6 denote sIgE levels categorized as high, very high, and extremely high, representing degrees of sensitization described as moderate to severe, severe, and extremely severe, respectively. Notably, the concentration of allergen-specific IgE indicates the degree of sensitization, but it does not serve as a proxy for clinical disease severity or diagnosis. Because participants with low levels of IgE sensitization (i.e., 0.35–2 kU/L) are less likely to have clinical FAs [17], our analysis specifically examined sIgE levels meeting or exceeding the “high” threshold criterion. Table 3 presents the age-stratified and sex-stratified distribution of high-level sIgE. Notably, children aged 1–3 years demonstrate the highest frequency of significantly elevated sensitization responses.

### 3.4. Gender Differences in Allergen Sensitization Profiles

Our analysis revealed significant gender disparities in food allergen sensitization patterns, with male participants demonstrating markedly higher positive rates than females for peanut, soybean, crustacean (crab and shrimp), and wheat allergens (Table 2). Interestingly, milk, codfish, egg, beef, and mutton exhibited no significant sex-based differences in sensitization rates. The male predominance persisted when examining moderate-to-severe reactions (levels 4–6), particularly for soybean, crustacean, and wheat allergens (Table 3).

**Figure 3:**
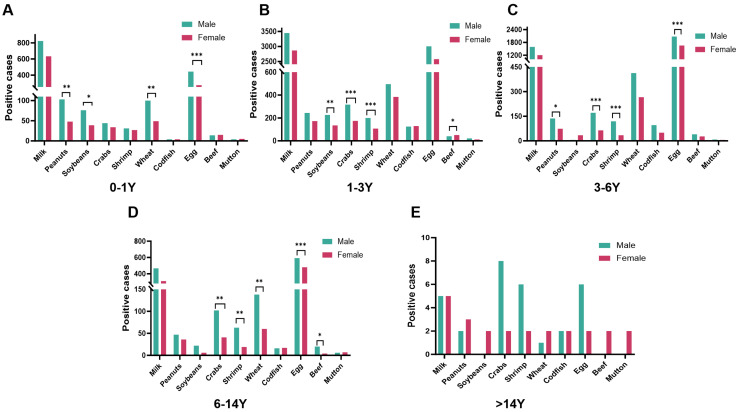
Sex difference in allergen sensitization in each age group. *: *p* < 0.05; **: *p* < 0.01; ***: *p* < 0.001.

The age-stratified analysis demonstrated consistent male-biased sensitization to major allergens like peanut, soybean, crab, shrimp, wheat and egg. Notably, distinct age-dependent patterns emerged—gender differences in soybean sensitization were present before 3 years of age, whereas differences for shrimp and crab appeared after the first year. Male participants also showed heightened sensitivity to peanut and wheat during infancy (0–1 year). For egg sensitization, males exhibited higher positivity rates than females across all subsequent developmental stages, with toddler period (1–3 years) being the sole exception (Figure 3). For the cohort of participants aged >14 years, the sample size was insufficient for conducting a reliable gender-stratified analysis, preventing definitive conclusions regarding adolescent sensitization patterns. These findings collectively suggest complex interactions between biological sexes and developmental stages and allergen-specific immune responses.

## 4. Discussion

This cross-sectional analysis systematically characterized allergen sensitization profiles and evaluated their age-stratified and sex-dependent variations among pediatric populations in the study region. We identified a pronounced heterogeneity in allergen sensitization profiles, with milk exhibiting the highest prevalence (31.18%), followed by eggs (30.57%), whereas mutton and beef exhibited minimal reactivity. Age-stratified analyses revealed critical developmental windows of sensitization vulnerability: toddlers (>1 and ≤3 years) exhibited peak IgE reactivity to milk, peanut, soybean, and seafood allergens, followed by a transitional shift to predominant egg, wheat, and beef sensitization during preschool years (>3 and ≤6 years), ultimately progressing to a dominance of mutton and crab allergens in school-aged children (>6 years). Notably, compared to females, males demonstrated significantly elevated sensitization rates (*p* < 0.05) for peanuts, soybeans, crustaceans and wheat.

Allergen-specific IgE testing serves as a cornerstone in vitro diagnostic tool for allergic disease assessments. The higher the sIgE level is, the stronger its correlation with the allergic disease [18]. The sIgE concentration objectively reflects the sensitization status. Importantly, the magnitude of sIgE reactivity exhibits limited correlations with clinical symptom severity. Moreover, detectable sIgE levels may occur in the absence of overt allergic manifestations, underscoring the distinction between immunological sensitization and clinical allergies [16]. A contemporary food allergy diagnosis fundamentally depends on clear clinical symptoms, while sIgE testing only indicates sensitization. And the oral food challenge persists as the diagnostic gold standard [19].

Our study found that milk and eggs are the two most common food allergens in the local pediatric sensitization profile. This pattern can be attributed to the fact that cow’s milk and hen’s eggs are typically among the first and most frequently consumed foreign proteins in the infant and toddler diet in this area. And the major allergenic proteins in milk (e.g., caseins) and eggs (e.g., ovalbumin and ovomucoid) are notably heat-stable and resistant to digestion [20]. This stability allows them to retain their immunogenic structure when reaching the intestinal immune system, thereby increasing their allergenic potential compared to more labile proteins. In line with our results, analyses based on pediatric populations in Shanghai, Eastern China, identified milk (18.1%) and eggs (17.8%) as the predominant food allergens [21], whereas prevalence studies from Dongying, Northern China, revealed eggs (33.04%) and wheat flour (19.13%) as the leading causative agents [22]. This geographical divergence in sensitization patterns likely reflects regional dietary practices, as Northern Chinese cuisine predominantly features wheat-based staples, such as noodles and steamed buns, whereas Shanghai’s dietary profile aligns more closely with that of our study region. These observations demonstrate that even within a single nation, regional variations in dietary exposure can significantly influence local allergen profiles. This underscores the necessity of conducting region-specific epidemiological assessments to inform accurate diagnostic and prevention strategies.

Compared with our team’s 2019 study of the same population using an identical sIgE detection system [23], the overall food sensitization prevalence increased from 44.83% (772/1722) (2019) to 48.12% (17,514/36,399) (2023–2024). This trend is consistent with the WHO’s surveillance data, which states that the vast majority of countries—such as Australia, Japan, South Korea, the United States, Norway, and China—have reported an increase in the prevalence of food allergies [8]. While milk sensitization rates remained relatively stable (33.15% to 31.18%), the prevalence of egg allergies demonstrated a statistically significant enhancement (24.56% to 30.57%). It is worth noting that the earlier 2019 cohort (*n* = 1722) represented a comparatively limited sample size, potentially introducing a selection bias when contrasted with the current comprehensive population-based survey (*n* = 36,399). This substantial scale difference underscores the greater statistical power and epidemiological reliability of our present findings.

Our data demonstrate a concordance with established epidemiological patterns, revealing that the peak allergic sensitization incidence occurs principally during early childhood (0–6 years) [21,22,24]. These sensitization patterns likely reflect vulnerabilities in early life, characterized by immature gastrointestinal barrier functions and an underdeveloped immunoregulatory capacity. During infancy, the developing epithelia, both mucosal and skin, are more leaky and prone to inflammatory responses [25]. Recent evidence reveals that tissue-resident interleukin (IL)-13- and IL-4-producing group 2 innate lymphoid cells (ILC2s) are enriched in human infant intestines compared to adult intestines [26]. Because ILC2s play a role in allergic reactions [27], their greater representation/activation in early life may contribute to a higher propensity in infants to develop allergic responses instead of immune tolerance.

Meanwhile, our study revealed significant gender differences, with male participants exhibiting higher sensitization rates and levels than their female counterparts. This is consistent with previous findings [21,22,23,24,28]. It appears that boys may be more vulnerable to allergen sensitization and more susceptible to food allergies. Although the mechanisms underlying sex differences in allergic reactions are not yet fully understood, it is widely accepted that sex is a biological variable that affects immune responses to both self and foreign antigens (for example, those from fungi, viruses, bacteria, parasites and allergens) [28,29,30]. Potential explanatory mechanisms encompass genetic mediators (notably immune-regulatory genes on the X chromosome), environmental factors (including nutrition and microbiota), and hormonal influences [29]. Many genes on the X chromosome regulate immune function and play an important role in modulating sex differences in the development of immune-related diseases [31]. For example, an experimental autoimmune encephalitis (EAE) and lupus model illustrated elevated Th2 cytokine levels (IL-4, IL-5, IL-13) in XY versus XX mice, independent of gonadal hormone effects [32]. This finding is particularly important considering that these cytokines are not only characteristic markers of Th2-type responses in food allergies but also play a dual role in both initiating and maintaining the process. Furthermore, accumulating evidence from animal models demonstrates significant sexual differences in the gut microbiota composition [33]. For instance, Lactobacillus plantarum and Bacteroides distasonis were enriched in B6 females as compared to B6 males, while Bifidobacterium was enriched in BALB/c females as compared to BALB/c males [34]. Since mucosal host–microbiome interactions are known to regulate dietary antigen sensitization [35], these microbiota differences likely underlie some of the observed sex-based variations in food allergen responses. While acknowledging hormonal contributions, our prepubescent cohort study design minimizes circulating sex hormone effects, suggesting their secondary role in observed sensitization disparities in this cohort. Further studies are needed to verify these hypotheses.

Several constraints merit careful consideration when interpreting these findings: first, the majority of participants were individuals with self-reported allergic conditions, potentially inflating sensitization estimates compared to regional population norms. Future investigations should prioritize community-level epidemiological surveillance to establish precise disease burden estimates—a critical dimension of public health research. Second, the allergen panel evaluated only the ten most prevalent food allergens, excluding other clinically relevant pediatric allergens (e.g., mango and tree nuts). Furthermore, as crude extracts were employed as the antigen source, the possibility of cross-reactivity cannot be ruled out entirely. Third, while we reported on basic demographic variables (age and sex), we acknowledge that the lack of more comprehensive clinical and demographic data is a significant limitation. Information such as detailed clinical histories (e.g., atopic comorbidities like eczema or asthma, family history of allergy, specific symptoms upon food exposure), dietary habits, geographic origins within Southwest China, and socioeconomic status was not systematically available. The absence of these variables restricts our ability to perform more nuanced analyses of risk factors associated with sensitization patterns and to fully characterize the clinical context of the sensitized cohort. A standardized prospective data collection process incorporating these additional parameters would strengthen future epidemiological investigations. Forth, the sensitization profiles presented in this study represent immunological risk markers rather than confirmatory allergy diagnoses—a fundamental caveat affecting the extrapolation of these epidemiological data to clinical decision-making or preventive health strategies. Last but not least, to avoid type II errors, this study did not apply multiple corrections, a decision that may increase the risk of false-positive findings. Future research should therefore employ more robust statistical methods.

Notwithstanding these limitations, this research represents significant progress. Building upon our prior research, the current cohort study demonstrates enhanced statistical power through its expanded sample size and elucidate significant sex-specific variations in allergic sensitization patterns. The longitudinal comparative analysis with historical data enables the identification of temporal trends in sensitization prevalence for major food allergens. Our findings can assist clinicians and public health officials in targeting effective interventions to specific pediatric groups and guide researchers toward further epidemiological studies of allergic diseases.

In summary, our investigation identified food allergen sensitization in 48.12% of pediatric participants within this region, indicating the urgent need for improved primary care physician training in allergy management. Crucially, our data delineate both the peak age of sensitization—revealing a critical window for preventive strategies—and significant gender-based disparities in susceptibility. These findings provide actionable demographic insights for public health initiatives, while the observed sex differences strongly advocate for gender-specific considerations in both risk assessment protocols and health education campaigns. Future studies would benefit from prospective cohort designs that combine advanced diagnostic tools (e.g., component-resolved diagnostics) with comprehensive clinical profiles (including symptoms, confirmed diagnoses, and family histories). This will be key to understanding sensitization trajectories and enabling precise, mechanism-based prevention.

## 5. Conclusions

Our data demonstrate a rising temporal trend in food allergen sensitization rates these years, with milk and egg representing the predominant allergenic sources. The observed sensitization profiles exhibit distinct variations across different age groups and between sexes. These findings provide valuable insights into the contemporary landscape of food allergen sensitization in the study region, although the underlying biological basis for the observed sex-based disparities remains to be elucidated through future investigation.

## Figures and Tables

**Table 1:** Characteristics of participants.

	Number of Participants	Percentage (%)
Age group		
0–1 Y	7538	20.71%
1–3 Y	14,238	39.12%
3–6 Y	8605	23.64%
6–14 Y	5047	13.87%
>14 Y	971	2.67%
Sex		
Male	20,467	56.23%
Female	15,932	43.77%
Month		
January	2384	6.55%
February	2796	7.68%
March	3386	9.30%
April	3588	9.86%
May	3576	9.82%
June	3466	9.52%
July	3714	10.20%
August	3556	9.77%
September	2455	6.74%
October	2257	6.20%
November	2195	6.03%
December	3026	8.31%
Total samples	36,399	

**Table 2:** Distribution of allergens in different age group and sex.

Allergen	Age Group [*n* (%)]					Sex [*n* (%)]		p*
	0–1 Y (*n* = 7538)	1–3 Y (*n* = 14,238)	3–6 Y (*n* = 8605)	6–14 Y (*n* = 5047)	>14 Y (*n* = 971)	Male (*n* = 20,467)	Female (*n* = 15,932)	
Milk	1457 (19.33%)	6313 (44.34%)	2794 (32.47%)	776 (15.38%)	10 (1.03%)	6334 (31.33%)	5016 (30.99%)	0.48
Peanut	151 (2.00%)	417 (2.93%)	210 (2.44%)	83 (1.64%)	5 (0.51%)	532 (2.63%)	334 (2.06%)	<0.001
Soybean	115 (1.53%)	362 (2.54%)	95 (1.10%)	28 (0.55%)	2 (0.21%)	386 (1.91%)	216 (1.33%)	<0.001
Crab	78 (1.03%)	491 (3.45%)	235 (2.73%)	143 (2.83%)	36 (3.71%)	642 (3.18%)	341 (2.11%)	<0.001
Shrimp	58 (0.77%)	307 (2.16%)	153 (1.78%)	82 (1.62%)	13 (1.34%)	419 (2.07%)	194 (1.20%)	<0.001
Wheat	149 (1.98%)	879 (6.17%)	679 (7.89%)	198 (3.92%)	5 (0.51%)	1147 (5.67%)	763 (4.71%)	<0.001
Codfish	8 (0.11%)	254 (1.78%)	146 (1.70%)	33 (0.65%)	2 (0.21%)	243 (1.20%)	200 (1.24%)	0.77
Egg	717 (9.51%)	5583 (39.21%)	3727 (43.31%)	1072 (21.24%)	28 (2.88%)	6120 (30.28%)	5007 (30.94%)	0.17
Beef	29 (0.38%)	91 (0.64%)	67 (0.78%)	24 (0.48%)	0 (0.00%)	114 (0.56%)	97 (0.60%)	0.68
Mutton	9 (0.12%)	34 (0.24%)	14 (0.16%)	13 (0.26%)	1 (0.10%)	40 (0.20%)	31 (0.19%)	0.91

* *p*-value were derived from comparison between the two gender groups.

**Table 3:** Distribution of high-level sIgE in different age and sex groups.

Allergen (4–6 Level)	Age Group (*n*)						Sex (*n*)		*p* *
	0–1 Y *n* = (7538)	1–3 Y *n* = (14,238)	3–6 Y *n* = (8605)	6–14 Y *n* = (5047)	>14 Y *n* = (971)	Total *n* = (36,399)	Male *n* = (20,467)	Female *n* = (15,932)	
Milk	23 (0.31%)	152 (1.07%)	50 (0.58%)	6 (0.12%)	0 (0.00%)	231 (0.63%)	115 (0.56%)	116 (0.73%)	0.05
Peanut	7 (0.09%)	26 (0.18%)	8 (0.09%)	2 (0.04%)	0 (0.00%)	43 (0.12%)	28 (0.14%)	15 (0.09%)	0.28
Soybean	10 (0.13%)	6 (0.04%)	0 (0.00%)	1 (0.02%)	0 (0.00%)	17 (0.05%)	15 (0.07%)	2 (0.01%)	<0.01
Crab	4 (0.05%)	78 (0.55%)	48 (0.56%)	31 (0.61%)	7 (0.72%)	168 (0.46%)	111 (0.54%)	57 (0.36%)	<0.05
Shrimp	3 (0.04%)	53 (0.37%)	24 (0.28%)	19 (0.38%)	6 (0.62%)	105 (0.29%)	71 (0.35%)	34 (0.21%)	<0.05
Wheat	10 (0.13%)	22 (0.15%)	7 (0.08%)	5 (0.10%)	0 (0.00%)	44 (0.12%)	32 (0.16%)	12 (0.08%)	<0.05
Codfish	0 (0.00%)	6 (0.04%)	3 (0.03%)	0 (0.00%)	0 (0.00%)	9 (0.02%)	6 (0.03%)	3 (0.02%)	0.74
Egg	24 (0.32%)	117 (0.82%)	50 (0.58%)	2 (0.04%)	1 (0.10%)	194 (0.53%)	100 (0.49%)	94 (0.59%)	0.19
Beef	0 (0.00%)	0 (0.00%)	0 (0.00%)	0 (0.00%)	0 (0.00%)	0 (0.00%)	0 (0.00%)	0 (0.00%)	0.99
Mutton	1 (0.01%)	2 (0.01%)	1 (0.01%)	1 (0.02%)	0 (0.00%)	5 (0.01%)	2 (0.01%)	3 (0.02%)	0.66

* *p*-value were derived from comparison between the two gender groups.

## Data Availability

The datasets used and/or analyzed during the current study are available from the corresponding author upon reasonable request.

## Abbreviations

The following abbreviations are used in this manuscript:

FA	Food allergy
NHANES	National Health and Nutrition Examination Survey
AA	African American
EA	European American
ELISA	Enzyme-linked immunosorbent assay
WAO	World Allergy Organization
ILC2	group 2 innate lymphoid cell
